# Developing new portals to safety for domestic abuse survivors in the context of the pandemic

**DOI:** 10.1111/hsc.14089

**Published:** 2022-10-21

**Authors:** Nicky Stanley, Helen Richardson Foster, Christine Barter, Claire Houghton, Franziska Meinck, Leah McCabe, Sarah Shorrock

**Affiliations:** ^1^ University of Central Lancashire Lancashire UK; ^2^ University of Edinburgh Edinburgh UK

**Keywords:** community touchpoints, COVID‐19, domestic abuse, pharmacies

## Abstract

This study examined the emergence and implementation of community touchpoints established in the UK during the COVID‐19 pandemic for victims/survivors of domestic abuse (DA). Community touchpoints are designated places, both online and in accessible settings such as pharmacies and banks, where victims/survivors can seek confidential advice and be directed to expert DA services. The research adopted a case study approach and explored a range of perspectives through expert interviews, document analysis, consultation with survivors and stakeholders and a survey of DA co‐ordinators. Four national community touchpoint schemes were identified and, of these, three were implemented rapidly and were available in 2020–2021 when the UK experienced lockdowns. Partnerships between Government/voluntary organisations and commercial businesses‐assisted design and implementation. Some stakeholders considered that the schemes lacked responsivity to the local context and noted challenges in providing a confidential service in rural areas. Whilst pharmacies, banks and online spaces were identified as non‐stigmatised and trusted places to seek advice, community touchpoints were judged less accessible for some groups including those experiencing digital poverty and victims whose movements were heavily scrutinised. Most of the touchpoint schemes targeted adults only. There were also concerns about whether frontline staff in commercial businesses received sufficient training. Whilst robust evidence of outcomes was limited, there were indications that the schemes had achieved good reach with some early evidence of take‐up. Testimonials indicated that victims/survivors were using the touchpoints in flexible ways which met their needs. Moreover, the wide reach and visibility of these initiatives delivered in non‐stigmatised settings may have served to raise public awareness of DA, reducing the silence that has traditionally surrounded it. Further research into the use and impact of these initiatives is required and there may be future potential to extend community touchpoints to include children and young people experiencing DA.

**What is known about this topic?**

- Community touchpoints are a recent initiative. They are designated places, both online and in pharmacies and banks, where domestic abuse (DA) victims/survivors can access advice and be directed to expert services.
- Whilst pharmacies have the potential to provide a frontline response to DA, concerns remain about the extent of training required.

**What this paper adds?**

- Under COVID‐19, public‐private partnerships enabled rapid roll‐out in the UK. Available online and in banks and pharmacies, touchpoints achieved wide reach.
- It is uncertain whether these initiatives respond to local contexts and whether they are equally accessible to all groups. Children are not targeted by most UK initiatives.
- Community touchpoints may have raised public awareness of DA.

## 1 DEVELOPING NEW PORTALS TO SAFETY FOR DOMESTIC ABUSE SURVIVORS IN THE CONTEXT OF THE PANDEMIC

The COVID‐19 pandemic has produced a rapid change in the way that public services are delivered and experienced. Whilst there have been restrictions and shortfalls in health and social care services, there has also been innovation with some planned developments rapidly accelerating and new partnerships emerging to deliver novel forms and configurations of services.

Early in the pandemic, domestic abuse (DA) services were vocal in raising concerns that DA rates would increase under restrictions that would confine victims/survivors and their abusers to the home whilst cutting off access to usual sources of support and advice (Home Affairs Committee, 2020; Refuge, 2020; Women's Aid, 2020a). The experience of confinement to the home appeared to strike an empathic note amongst many organisations and individuals not previously engaged in providing support or services to DA victims and DA services received contributions and offers of support from new sources (Stanley et al., 2021).

This paper reports on the development and implementation of community touchpoints: these are new initiatives that emerged in the UK in the context of the pandemic. Touchpoints are defined as safe spaces or portals from which victims/survivors can seek information and access expert help. They are non‐stigmatised, easily accessed spaces with either a community or online presence. Most of the touchpoint schemes considered here are the product of partnerships involving the independent DA sector, professional organisations, commercial organisations and/or Government. They share a focus on place or space whilst also drawing on concepts of community capacity and accessibility.

We used a case study approach to examine the design and development of these schemes and aimed to identify their strengths and limitations. We also draw out recommendations and messages for embedding and sustaining these initiatives in the longer term.

### 1.1 Background

In developing services, the DA sector has struggled to reconcile the principles of safety and accessibility. The safety of victims has been a core principle informing provision from the earliest days when refugees were first introduced (Hague, 2021). However, a focus on safety has entailed secrecy with the locations of refugees disguised and with DA services lacking a public face and often a community presence (Haaken & Yragui, 2003). This has proved frustrating at times for those seeking to access or use services (Stanley, 2015; McCarry et al., 2018; Bracewell et al., 2020). Campaigns have attempted to address this problem but the lack of local visibility remains an issue for many DA services.

The National Commission on Domestic and Sexual Violence and Multiple Disadvantage established by AVA (Against Violence & Abuse) and Agenda, two UK DA organisations, recognised the need to improve access to services and advocated for approaches such as ‘no wrong door models’ and one‐stop shops for the DA sector (AVA & Agenda, 2019). A report from the House of Commons Home Affairs Committee (2018) built on NICE (2014) guidance in arguing that other public services needed to be equipped with the skills and guidance that would enable frontline staff to direct DA victims/survivors to specialist DA services:

More training, central guidance and national oversight is required to ensure that public sector staff dealing with members of the public can identify signs of domestic abuse, respond appropriately and know how to help victims of domestic abuse access whatever specialist support they may need. (para 50, House of Commons Home Affairs Committee, 2018)

The community touchpoint schemes described below offer an alternative means by which DA services could increase their accessibility. They aim to address the lack of community visibility by providing access to specialist DA support and services in spaces that are well‐used but dedicated to other purposes. In this sense, access to DA support is ‘hidden in plain sight’ and non‐stigmatised. The spaces chosen as community touchpoints are mostly places where the public can access various forms of advice and assistance, including health and financial advice: they are sites of expertise and professional activity and this, together with their identity as places that everyone can enter and use, contributes to their status as safe but accessible places.

Pharmacies were amongst the first sites to be recruited as community touchpoints. Their potential for providing access to DA services has already been identified both in the US and the UK (Cerulli et al., 2019; Lewis et al., 2018) and Lewis et al.’s (2021) study involving interviews with 20 pharmacists undertaken prior to the pandemic found a readiness to contribute to the delivery of public health services and highlighted the opportunities offered by the accessibility and inclusivity of pharmacies. However, during the pandemic, the settings harnessed as community touchpoints extended beyond health settings into commercial and digital spaces.

## 2 METHODS

This research was undertaken as part of an international study that explored the response to DA during the pandemic in four countries: the UK, Australia, Ireland and South Africa. Completed between November 2020 and June 2022, the research included mapping studies undertaken in each country and four in‐depth case studies that permitted deep dives into new initiatives (see www.Dahlia19study.com). These initiatives were selected on the basis of consultation with stakeholders and analysis undertaken in the course of the mapping studies.

The UK case study utilised data collected for the mapping study which included: the analysis of 180 relevant reports and documents identified with the assistance of a widely distributed call for evidence, online searches and consultation; a survey of 31 DA co‐ordinators in England and Wales; 24 semi‐structured interviews with DA experts across the UK and four consultation groups and interactive webinars with key stakeholders. Four survivors' advisory groups were also held in England and Wales. These were recruited and convened with the assistance of DA organisations that were available to provide support for any participating survivor who might require it. Additional data were collected for the deep dive afforded by the case study approach (Yin, 2009). This comprised online documents and grey literature relating to DA community touchpoints accessed between July and December 2021. An additional eight semi‐structured interviews with professionals with experience in the design and implementation of community touchpoints were completed in early 2022. All interviews took place online or by phone with interviewees representing DA organisations, devolved governments, the police and public health and pharmaceutical professionals. Interviews were transcribed and coded. All data were analysed against a framework consisting of key questions developed to elucidate the reach, acceptability, accessibility, impact, barriers to implementation, recommendation for strengthening and future promise of these interventions.

Ethical approval for the study was acquired from the Universities of Central Lancashire and Edinburgh.

### 2.1 Limitations

The research was completed during the pandemic at a time when the initiatives studied were newly implemented and there was little robust data available in respect of impact. Whilst the study took place at a ‘moment of crisis’ when there were opportunities to capture change and innovation, there were reduced opportunities for interventions to embed and mature. The research team was in part reliant on existing data which reduced the extent and quality of data available for analysis. Future research could usefully examine the impact and study these schemes over a longer period.

## 3 RESULTS

### 3.1 The community touchpoint schemes

**FIGURE 1:**
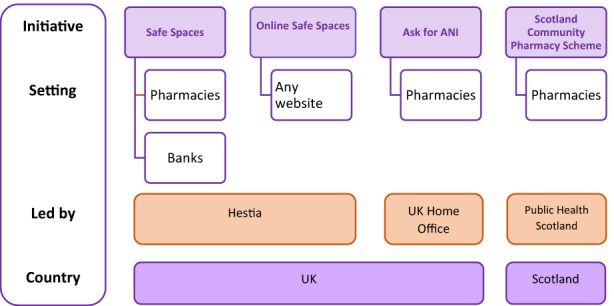
Key national community touchpoint schemes.

This study identified four community touchpoint schemes, all aspiring to national reach and initiated during the pandemic. The Mascarilla‐19 (Mask‐19) codeword scheme introduced by the Institute of Equality in Spain's Canary Islands (World Bank, 2021) appears to have been highly influential as a model for pharmacy‐based responses to Covid‐19 restrictions, but the schemes outlined briefly below extended into other spaces. Figure 1 identifies the key UK community touchpoint schemes described further below.

1. *Safe Spaces* was developed by Hestia/UK Says No More, a London‐based DA organisation, in partnership with four large pharmacy chains, the General Pharmaceutical Council and the Royal Pharmaceutical Society. Launched in May 2020 following a pilot in one London borough, the scheme initially recruited pharmacies in England that were prepared to enable their consultation space to be used by DA survivors. Survivors would be given access to a telephone and provided with the national DA helpline number, as well as details of Hestia's app and information about their local DA service. The scheme was extended in May 2021 to include TSB bank branches and by early 2022, additional funding had been accessed which was to be used to expand the scheme across Scotland and Wales and another bank (HSBC) joined in April 2022.
2. Also in 2020, Hestia initiated *Online Safe Spaces* which took the form of a logo button/widget located on a wide range of commercial and Government websites. This allowed users to access DA information online via a portal which ensured their internet use was not tracked. This scheme involved the Royal Mail Group and Hawkrose, a management consultancy, who together donated the necessary technology. *Online Safe Spaces* was initially available via the Royal Mail, Parcelforce and six other websites and, by March 2022, was displayed on the websites of 64 UK organisations, including some Government departments (Hestia, 2022a).
3. *Ask for ANI* (action needed immediately) was launched by the Home Office in January 2021 and is the scheme that most closely resembles the Mascarilla‐19 initiative. ANI is an emergency code word that DA survivors use in a pharmacy to signal their need for help, and staff will then provide confidential and safe assistance enabling them to contact the police or support helplines. The scheme was developed in collaboration with SafeLives (2021) and involved survivors in the design and development of promotional and training materials; other partners included the Metropolitan Police and Women's Aid. Hestia took over the operational running of the scheme from the Home Office in the Spring of 2022.
4. The Scottish *Improving Community Pharmacies' Response to Rape and Sexual Assault* scheme has a different remit but is included as it provides an interesting contrast. It differs from the voluntary schemes described above, as it is envisaged that participation will become part of NHS contractual arrangements for Scottish pharmacies should early implementation prove successful. The initiative is targeted at adult victims/survivors of rape and sexual assault, specifically women and those accessing a prescription for emergency contraception; however, young people may be included as emergency contraception can be prescribed to people over 12 years. Funded by the Scottish Government for 2 years from December 2021, the scheme is led by Public Health Scotland. At the time of writing, the scheme is in the first phase of a national rollout.

Key themes identified included: rapid implementation; responsivity to local contexts; training for frontline staff; reach and uptake; access for minority groups and for children and young people and awareness raising. These are discussed below.

### 3.2 Rapid implementation

Participants generally agreed that *Ask for ANI, Safe Spaces* and *Online Safe Spaces* had been speedily developed and established. Those administering Hestia's schemes had: ‘no idea that they would be able to turn around something like that as quickly as they did. I didn't … know our team could do that.’ (Case Study interview 2). Rapid roll‐out of touchpoints during 2020–2021 meant that they were available during lockdowns. The crisis situation acted to free up some resources by suspending normal practices: for instance, General Pharmaceutical Council inspectors had been able to promote the *Safe Spaces* scheme due to the temporary suspension of their inspections during the lockdown. The cost‐free involvement of some large commercial organisations also provided extra capacity.

Those planning and delivering the touchpoint schemes were able to draw on existing networks and partnerships to assist in planning and roll out. For example, *Ask for ANI* was promoted by the police:

What we did was do the push of driving it, ensuring that across London our partners knew about it, our police colleagues, you know, our community support officers knew about it, and we did that. We put it in a box folder for them, so they had access to the PDF material directly, you know, obviously lots of coms went out. (Case Study interview 7)

However, new public‐private partnerships were also forged and rapidly utilised. These may have been stimulated by increased empathy for DA victims fostered by COVID‐19 restrictions and by a readiness to use private expertise for the public good in a time of crisis.

Despite the drive for rapid roll‐out, DA survivors were consulted on the development of *Ask for ANI's* promotional materials and training. This consultation was facilitated by SafeLives, a specialist DA organisation which supports an established group of DA survivors, described as *Pioneers*, who have contributed to the design and planning of a range of DA services (Tomlinson, 2021).

In contrast, implementation of the Scottish pharmacies scheme was much slower: the scheme was first proposed early in 2020 but funding was not available for pilot start‐up until December 2021. However, the extended planning period allowed for the development of national governance and accountability structures and partnership buy‐in.

### 3.3 Responsivity to local contexts

Hestia's *Safe Spaces* was the only one of the established schemes that grew from a pilot. It was notable that the other community touchpoint initiatives were initiated by the central government adopting an international initiative (*Ask for ANI)* or sprang from a national collaboration between commercial and independent sectors (Online Safe Spaces). These origins may have contributed to a perception of these interventions as top‐down and, whilst community touchpoints were generally welcomed by research participants, concerns were voiced about the degree to which they were responsive to different UK jurisdictions and local settings. Respondents from Northern Ireland felt that *Ask for ANI* was ‘*forced upon’* the nation by Westminster with little consideration of the Northern Irish context, and some of those consulted in Scotland were similarly critical, with several pointing out that an English DA helpline had been advertised on *Ask for ANI* posters in Scotland instead of Scotland's Domestic Abuse and Forced Marriage Helpline.

Whilst participants agreed that pharmacies or banks were generally places that women could potentially access without raising suspicion, some questioned whether businesses in rural areas could offer sufficient confidentiality and safety:

most of our chemists would have local people working in it … So again, that's how anonymous are you actually going in and if you go into the side room, which is unusual activity, what people think you're actually there for, so I still think it's quite public … and the chances are you're going to know who's serving behind. Are you going to be in earshot of somebody? (Case Study interview 5)

Similar concerns were voiced by participants from independent pharmacies in Lewis et al.’s (2021) study.

Linking touchpoints with local DA services was viewed as a means of ensuring local relevance and congruency and those developing the community pharmacy scheme in Scotland emphasised the importance of working with gender‐based violence leads based in the NHS boards and with violence against women (VAW) partnerships in each local authority. This respondent highlighted the value of the initiative linking to both local and national services:

… we also wanted it to have a really clear link to local services as well, rather than just the national helplines. Because I suppose we recognised the opportunity that it gave to signpost people on to a range of services …. (Case Study interview 1)

The virtual location of *Online Safe Spaces* allowed this scheme to transcend the specificity of particular places or settings. This touchpoint was seen as highly accessible and interviewees noted that it offered an additional flexible route to support that victims/survivors would appreciate:

It's giving them choice, it's giving them options. It's giving them resources so that they can… be empowered to make choices and supported to do that … I go back to Online Safe Spaces and the fact that we could put that everywhere and … why not? (Case Study interview 3)

### 3.4 Training for Frontline Staff

Those implementing the touchpoint schemes emphasised that frontline staff received DA training including handling disclosures. Pharmacists already receive level 2 safeguarding training that equips them in the basics of protecting vulnerable adults and children and this was judged to provide them with the minimum level of knowledge and confidence required to respond effectively (Case Study interview 3). Hestia provided an e‐toolkit for participating pharmacies and one partner (Boots) provided additional training for pharmacists, noting that their role was to offer a safe space and available support and signpost DA victims to relevant services rather than functioning as DA professionals. Bank staff completed a 60‐minute e‐learning course. In common with participants in Lewis et al.’s (2021) study, a participant from the pharmacy sector commented that staff were worried [whether] they'd have the skills to deal with people … or that they might do the wrong thing.’ (Case Study interview 8). A DA organisation in Northern Ireland created and delivered a virtual training programme for pharmacies as many had simply ‘put the poster up and whatever, but they hadn't thought.’ (Interview 11, NI).

Delivering the training to large numbers of staff was experienced as demanding at a time of staff shortages, PPI and social distancing requirements. However, implementation appeared to have been assisted by high levels of enthusiasm for and engagement with the touchpoint schemes:

[pharmacies] were delivering core services … obviously there was all the cleaning requirements … it did require a little bit of training, during a time when, quite frankly, it was a miracle that they kept going. So it involved a commitment and ownership by the staff. I know Boots in particular, they didn't force it on everybody, and a lot of people … came forward and said it's the right thing to do.’ (Case Study interview 8)

Some respondents questioned whether the limited contact with the frontline bank or pharmacy staff could provide the necessary support over time that might be required for disclosure: ‘We know that disclosure is difficult and that very often, creating the circumstances for women to disclose requires more than a poster saying, there's a safe space here if you need it.’ (Interview 7, Scotland)

### 3.5 Reach and Take‐Up

Whilst limited evidence was available in respect of outcomes for DA survivors using these schemes, documentary analysis provided evidence on take‐up and organisational and geographical reach. There were also some anecdotal testimonies available. The account below provides a ‘success story’ in respect of Hestia's *Safe Spaces* initiative:

She went to the safe space when she went to get her prescription. … her first phone call was to her family … she used it I think a further six times and eventually fled and left the relationship. And was able to use it to access local support, IDVA, legal support, and eventually, so put everything in motion to leave’. (Case Study interview 3)

This account challenges the suggestion above that community touchpoints cannot provide support over time and provides an example of a survivor transforming a help‐seeking event into a process. As another informant noted:

‘It's [a] safe space that people can return to at different points if they need to. Because we know with domestic abuse, people don't just call, get help and then leave and that's it … it's a cycle and it can take a long time for change to happen and for people to get that support that they need and that confidence’ (Case Study interview 4)

In November 2020, Hestia reported that 4171 pharmacies were participating in the *Safe Spaces* scheme, these were mainly located in England (82%) with 10% in Scotland, 4% in Wales and 3% in Northern Ireland (Hestia, 2020). Participating pharmacies primarily belonged to one of the four major chains with only between 1% and 15% of participating pharmacies located in the independent pharmacy sector in each country of the UK. The TSB bank joined the scheme in 2021 and, by March 2022, 5720 pharmacies and 290 bank branches across the UK had signed up for *Safe Spaces* (Hestia, 2022a). This was anticipated to increase to 800 branches in April 2022 when HSBC joined the initiative (Hestia 2022b).

Case study interviewees expressed concerns that the *Safe Spaces* scheme was not reaching more remote rural areas: in Scotland, it was felt to be largely confined to the central belt of the country, whilst in Wales, some rural areas were felt to be ‘missing out’: ‘rural areas in Wales…that didn't fit into that, you know, that kind of [national] requirement…there's a really good spread of Safe Spaces but there are some definite little tiny pockets where we would like more.’ (Case Study interview 3). These issues had been recognised and potentially addressed by the appointment of two coordinators for Wales and Scotland to work on increasing reach, in particular liaising with local networks able to support women appropriately in rural and remote locations.

By November 2021, the *Online Safe Spaces* scheme had been adopted by 45 organisations, resulting in 700,000 online visits. This had increased to 64 organisations by March 2022, including 10 rail companies, with 934,000 individual hits recorded online (Hestia, 2022a).

The Home Office was unable to provide detailed findings from the commissioned evaluation of *Ask for ANI* and we are reliant on headline data available via a press release in January 2022 (Hestia 2022b). This reported that the scheme had attracted ‘almost 100’ recorded disclosures following the use of the code word and that: 95% of individuals who had asked for ANI then used *Safe Spaces*; 14% were supported by a pharmacist to dial 999 and 8% were supported to make a non‐emergency 101 call to the police.

Most of the 31 DA coordinators responding to our survey in June 2021 were employed by local authorities with a few working for the regional Crime Commissioner's office or in the independent sector. Thirty per cent reported some or good take‐up of pharmacy codeword schemes and 41% reported some or good take up of *Safe Spaces* schemes in their region. However, over a third of respondents did not know or had not heard of either initiative.

Some of those interviewed reported a lack of clarity as to which and how many pharmacies were participating in the *Ask for ANI* scheme and this confusion may have been heightened by the potential for confusion between this scheme and Hestia's Safe Spaces. However, one participant noted the wide coverage associated with a large retail pharmacy chain, such as Boots, which was a household name, delivering *Ask for ANI*, and commented that the ‘door is now open’ for further collaborations (Interview 7, Scotland).

### 3.6 Access for minority groups and children and young people

There were early queries as to whether codeword schemes were sufficiently accessible for marginalised groups and for those facing the most barriers to support, namely Black and Minoritised women, migrant women, deaf and disabled women and LGBT+ survivors (Women's Aid, 2020b). Promotional material was available in other languages via Hestia and Home Office websites but there were concerns that it was not always displayed at touchpoints. Hestia's app and website had the built‐in facility to provide information in multiple languages.

In terms of the spaces utilised, online spaces were considered to be accessible to many groups although it was noted that digital poverty could prove a barrier for some DA victims/survivors. Pharmacies were identified by campaigning groups as accessible spaces for older adults (Hourglass 2020), and as places that were likely to be fitted with hearing loops. Some participants noted that women from some Black and Minoritised groups were less likely to be able to access public places such as pharmacies or banks on their own and that refugee women might have concerns about revealing their insecure status. Comments about the high levels of scrutiny in some rural communities have been noted above. In contrast, the community pharmacy scheme in Scotland aimed to ensure that the service was accessible to marginalised groups, specifically women who sell sex, and survivors with drug and alcohol problems, and core training had been designed to this end.

With the exception of the Scottish pharmacy scheme which had developed specific procedures to support young people seeking emergency contraception, the community touchpoint schemes largely targeted adults. However, the need for safe spaces for children and young people living with DA during the pandemic was heightened due to their ‘invisibility’ to both mainstream and specialist services (Chevous et al., 2020; Stanley et al., 2021, Morrison & Houghton, 2022). Some regrets were expressed by interviewees about the failure to provide touchpoints for children and young people and there was a suggestion that schemes might be extended to include them in future:

So we're still at the place of having it targeted for adults successfully … Yes, it's something we'd be open to I think, once we get that promotion around Scotland really up and running (Case Study interview 4)

### 3.7 Awareness raising

Those consulted noted that community touchpoints had contributed to raising public awareness of DA during the pandemic, particularly through the involvement of large, public‐facing companies. The presence of *Online Safe Spaces* on commercial websites served a similar function: ‘any publicity, anything that's going on around safe spaces, people putting online safe spaces, our work with businesses. It's making it a societal issue’. (Case Study interview 3).

The partnerships built with high‐profile public and commercial organisations were also considered valuable in that awareness of DA and the touchpoint scheme reached large numbers of their employees:

Royal Mail employs 136,000 people. So, when you do the figures ‐ is it one in four women, one in six men ‐ you come out at around 25,000 employees will have or are experiencing it … And actually, so this widget is on both Royal Mail's intranet and external website, and that's the same for everyone. (Case Study interview 2)

## 4 DISCUSSION

Community touchpoint schemes have identified and publicised safe and destigmatised spaces in the community where DA survivors can access advice and information and, in doing so, they have made domestic abuse and DA services more visible at the community level. The physical locations chosen as touchpoints are, for the most part, businesses such as pharmacies and banks, where expertise (although not necessarily DA expertise) is known to reside and which are established and trusted sources of advice. The online spaces utilised have encompassed a wider range of large organisations, all with a strong public identity.

Disclosing and seeking support for DA is frequently understood as a process rather than an event and informants were concerned that brief one‐off contacts with staff who had received limited training in responding to DA would fail to offer the continuity and security provided by an existing relationship with a friend or family member or by a specialist practitioner. However, there were some early indications of survivors adapting the scheme to provide a service over time that kept pace with their needs. Online services can of course be visited at whatever pace fits an individual's needs and situation and, in this sense, the touchpoint schemes offered a considerable amount of control to those using them. These place‐based community touchpoints contrast sharply with other schemes that train and support community‐based volunteers or ambassadors to receive and respond to DA disclosures: for example, Women's Aid *Ask Me* initiative provides community volunteers with one or two full days of training and follow‐up support (Public Health England, 2021; Stanley et al., 2021). However, harnessing large chains of pharmacies and banks did allow the touchpoint schemes to achieve high reach. Further research and longer‐term evaluation are required to examine the range of ways in which touchpoints have been used and the outcomes for those who have used them.

Some groups of DA victims/survivors may have found community touchpoints to be less accessible than others and informants highlighted the potential for those women subject to high levels of scrutiny and those who experience digital poverty to be excluded. Leigh et al. (2022) argue that engagement with a diverse range of stakeholder groups is essential in identifying the needs of the most vulnerable DA survivors and some consultation with DA survivors was built into the early stages of these schemes. However, extensive consultation with a variety of groups may have proved difficult in the drive for rapid implementation. There remain opportunities for these schemes to be refined and developed through consultation with relevant survivor groups in order to ensure their accessibility for marginalised groups.These interventions have been built on partnerships fuelled by the altruism and empathy that a global pandemic has triggered. There are plans for the schemes to be extended and strengthened across Wales and Scotland and this, together with the transfer of the management of *Ask for ANI* from the Home Office to Hestia should increase reach and reduce duplication. It will be interesting to learn whether these partnerships continue to thrive in a post‐pandemic context. Some participants envisaged extending community touchpoints to additional settings:

we're moving to integrated care systems and whole population views within an area, and they probably need to move so that it is part of a whole systems approach. That, yes, people can go into a pharmacy or they could go into their GP or it could be a dentist … (Case Study interview 8)

## 5 CONCLUSION

The restrictions imposed during the pandemic required those looking to maintain and increase the availability of DA services to think ‘outside the box’ and identify new ways in which information and services could be accessed. The initiatives described here significantly broadened the range of settings and organisations involved in linking those in need of support to services, moving beyond the third sector and public services into the commercial sector. Likewise, the use of online spaces extended opportunities for DA victims/survivors to access advice and information about services safely and at a pace and in a manner that suited them.

There were indications of gains in respect of awareness raising which occurred when services were advertised and available in places beyond the usual realms of health and social care in a wider range of community settings. The use of such settings may contribute to eroding the silence that has previously surrounded DA and future evaluations might usefully explore any attitudinal changes associated with community touchpoints.

Questions were raised by experts interviewed about the extent and availability of training provided for those on the frontline and it was reported that some of the community touchpoints were more accessible to some groups of victims/survivors than to others. In particular, only the Scottish scheme for victims of rape and sexual assault was accessible to some groups of young people. Given concerns about the ‘invisibility’ of children and young people living with DA during the pandemic, there may be potential for considering how touchpoint schemes could be adapted and made accessible to children and young people both during future crises and as part of the broader service landscape more generally.

## AUTHOR CONTRIBUTIONS

All authors contributed to the conception and design of this study. All authors were involved in data collection and analysis. NS prepared the original draft with assistance from HRF. All authors have provided critical feedback on the manuscript and have read and agreed to the published version.

## CONFLICT OF INTEREST

The authors have no conflicts of interest.

## Data Availability

The data that support the findings of this study are available from the UK Data Service Repository with the agreement of the lead author.
